# Preoperative HO-1 Levels as Prognostic Factor for Adverse Cardiac Events in Elder Patients Undergoing Non-Cardiac Surgery

**DOI:** 10.1371/journal.pone.0058567

**Published:** 2013-03-19

**Authors:** Hong Zheng, Hai-Ping Ma, Jiang Wang, Ming Ma

**Affiliations:** Department of Anesthesiology, the First Affiliated Hospital of Xinjiang Medical University, Urumqi, China; The University of Tennessee Health Science Center, United States of America

## Abstract

**Background:**

Hypoxia-inducible factor-1α (HIF-1α) and heme oxygenase-1(HO-1) are involved in the tissue hypoxic response.

**Hypothesis:**

HIF-1α and HO-1 levels may predict cardiac ischemia and adverse cardiac events during non-cardiac surgery.

**Methods:**

HIF-1α and HO-1 levels were determined in elderly patients undergoing non-cardiac surgery preoperatively and at 30 minutes, 48 and 72 hours postoperatively. Results were analyzed with respect to the occurrence of adverse cardiac events.

**Results:**

A total of 380 patients with a mean age of 65.3 years were included, and 54 (14.2%) who had adverse cardiac events during or after the surgery. HIF-1α and HO-1 levels in the adverse cardiac event group were significantly higher than in the group without adverse cardiac events at each time point (all, *P*<0.05). In multivariates regression analysis, the odds of an adverse cardiac event was increased by every 1-year increase in age (odd ratio [OR] 1.39, *P*<0.001), abnormal ECG at baseline (OR 2.27, *P* = 0.048), myocardial infarction history (OR 3.18, *P* = 0.015), and positive baseline cTnI level were associated with an increased likelihood of an adverse cardiac event (OR 8.78, *P* = 0.019), and for every 1 unit increase of HO-1, the odds of an adverse cardiac event increased by 1.30 (*P* = 0.002).

**Conclusion:**

Determination of preoperative HO-1 levels may aid in identifying patients at risk of developing ischemic cardiac events.

## Introduction

With the increasing age of the population, more elderly patients with coronary heart disease (CHD) are undergoing non-cardiac surgery leading to an increase in the incidence of perioperative cardiac-related adverse events [1], [2]. The nature of adverse cardiac events is myocardial ischemia and hypoxia, and studies have shown that hypoxia inducible factor (HIF) is the most important transcription factor maintaining oxygen homeostasis in mammalian body tissues [3]–[6]. HIF-1 is present in the heterodimer form and consists of an α subunit (HIF-1α) and β subunit (HIF-1β). HIF-1α is the hypoxia-dependent subunit of HIF, which is highly sensitive and specific to the hypoxia response [5]. HIF-1α is present in the cytoplasm and rapidly degraded under normoxic conditions, and it exerts the biological effects via stabilization and nuclear translocation to form a functional complex with intranuclear HIF-1β in nucleus. It regulates the expressions of a series of hypoxia-related genes when hypoxia occurs [5], [6]. The other protein important for hypoxia response, Heme oxygenase-1 (HO-1), catalyzes the degradation of heme to carbon monoxide, iron, and biliverdin [7]. HO-1 expression is directly mediated by the HIF-1, and activation of HIF-1α/HO-1 has a protective role against acute coronary ischemia under hypoxic conditions [3]. As a marker of acute tissue hypoxia [4], HIF-1α has been shown to have a strong predictive value for myocardial cell apoptosis [8].

Thus, the purpose of this study was to determine the predictive value of preoperative HIF-1α and HO-1 levels for the development of adverse cardiac events in patients undergoing non-cardiac surgery.

## Patients and Methods

### Patients

Patients with moderate and high-risk CHD who underwent elective non-cardiac surgery at the First Affiliated Hospital of Xinjiang Medical University between January 2010 and March 2012 were included. This study was approved by the Ethics Committee of the hospital (approval number: 20101215), and all patients provided written informed consent.

Criteria for inclusion were: 1) Age 61–80 years; 2) Undergoing elective moderate to high-risk non-cardiac surgery [9] (e.g., liver, gallbladder, urinary, gastrointestinal, and gynecological surgery); 3) Meeting the 1979 World Health Orgaization (WHO) CHD diagnostic criteria and the CHD diagnosis and treatment guidelines developed by the Chinese Society of Cardiology, the Chinese Medical Association in 2001 [10]; 4) Complete preoperative cardiac function assessment and New York Heart Association (NYHA) Functional Class I–III [11]; and 5) Normal preoperative liver, lung, and kidney function. The exclusion criteria were: 1) Emergent and low-risk surgery; 2) Severe preoperative infection and/or liver, spleen, kidney, and lung dysfunction; 3) Congenital heart disease, cardiomyopathy, rheumatic heart disease, pulmonary heart disease, severe heart failure, and severe arrhythmia; 4) Inability to cooperate (e.g., mental disorder, disturbance of consciousness, and mental retardation); and 5) The presence of infectious disease (hepatitis B, hepatitis C, syphilis, and AIDS).

### Blood collection and testing

Blood samples were collected via the cubital vein on the morning of surgery, 30 min and 48 and 72 hours after surgery. One 7 mL venous blood sample was collected and 4.0 mL sodium heparin was added for anticoagulation. Four milliliters of whole blood was used for the detection of cardiac troponin I (cTnI), and the remaining 3 mL was allowed to stand at room temperature for 30 min, and then it was centrifuged at 3,000 rpm at a temperature below 4°C for 15 min. The serum was collected and stored at −80°C until analysis.

A double antibody sandwich enzyme-linked immunosorbent assay (ELISA) was used for detection of HIF-1α and HO-1. The kit was purchased from Shanghai Sun Biological Engineering Co., Ltd., and the assays were performed according to the manufacturer's instructions. A microplate reader (Bio-Rad3550 type) was used to measure the optical density at 450 nm, and serum HIF-1α and HO-1 levels were calculated from the standard curve. cTnI levels were detected using the ACCESS cTnI assay (Beckman Coulter, Inc., Chaska, MN) as instructed by the user manual.

### Adverse cardiac events

Diagnostic criteria of adverse cardiac events were as follows: 1) Acute myocardial ischemia: horizontal ST segment or ST segment depression ≥1 mm or ST segment elevation ≥2 mm 80 ms after the J-point; 2) Acute myocardial infarction: precordial pain >30 min with ECG findings of new Q waves, ST segment depression or arched elevation, cTnI >3.1 g/L; 3) Malignant arrhythmia: atrial fibrillation, paroxysmal supraventricular tachycardia, and ventricular premature beats with obvious symptoms or altered hemodynamics that require drug treatment; 4) Congestive heart failure: shortness of breath, jugular vein distention, gallop, chest X-ray shows pulmonary edema; 5) Cardiac death: death caused by myocardial infarction, heart failure, and arrhythmias. A 12-lead ECG was performed at the time of blood sample collection, and as required clinically.

### Outcome measurements

Primary outcome measurements were serum HIF-1α and HO-1 levels. Secondary outcome measurements were 12-lead ECG and troponin levels (cTnI). Other outcome measurements studied were intraoperative ST-segment analysis, blood pressure, heart rate and SpO2, end-tidal CO2, amount of intraoperative blood loss, volume of fluid infusion, and urine output.

### Statistical analysis

Continuous data were presented as mean ± standard deviation (SD), categorical data were presented as count and percentage. For the comparisons between the patients with and without cardiac adverse event, the 2 independent samples t-test and Fisher's exact test were performed for continuous and categorical data, respectively. Logistic regression analysis was performed to evaluate factors associated with adverse cardiac events. Factors that obtained statistical significance in univariable regression analyses and had no obvious collinearity were entered into the multivariable logistic regression analysis. The cTnI positive rates in various cardiac adverse event groups were compared by the Fisher's exact test. All statistical assessments were 2-sided and considered as significant when *P*<0.05. Statistical analyses were performed using SPSS 15.0 statistics software (SPSS Inc, Chicago, IL, USA).

## Results

### Patients

During the period from January 2010 to March 2012, 380 patients who received non-cardiac surgery were enrolled. The patients included 176 males and 204 females, with a mean age of 65.3 years. There were 54 (14.2%) who had adverse cardiac events during or after the surgery, and 40 (74.1%) had the event within 3 days after surgery.

### Associations of adverse cardiac events and patient baseline characteristics

A comparison of the characteristics of the patients with and without adverse cardiac events is shown in Table 1. The patients with adverse cardiac events were significantly older than those without cardiac adverse event (67.0 vs. 65.1 y, *P*<0.001), were more likely to be smokers (37.0% vs. 23.9%, *P* = 0.045), be ASA grade [12], [13] III (35.2% vs. 18.1%, *P* = 0.006), have a history of myocardial infarction (24.1% vs. 9.5%, *P* = 0.005), and less likely to be of Han ancestry (46.3% vs. 64.4%, *P* = 0.015). The baseline levels of cTnI, HIF-1a, and HO-1 were higher in the adverse cardiac event group than in the group without cardiac adverse event (cTnI positive rate: 9.3% vs. 1.8%, *P* = 0.011; HIF-1α: 38.9 vs. 33.7 ng/mL, *P* = 0.002; HO-1: 32.3 vs. 27.4 ng/mL, *P*<0.001).

**Table 1:** Characteristics of patients with and without adverse cardiac events.

		Adverse Cardiac Event (n = 54)	No Adverse Cardiac Event (n = 326)	P-value
Age (y)		67.0±3.0	65.1±2.5	<0.001*
Gender	Male	31 (57.4)	145 (44.5)	0.104
	Female	23 (42.6)	181 (55.5)	
Race	Han	25 (46.3)	210 (64.4)	0.015*
	Other	29 (53.7)	116 (35.6)	
NYHA class	I	19 (35.2)	164 (50.3)	0.111
	II	27 (50.0)	126 (38.7)	
	III	8 (14.8)	36 (11.0)	
ASA grade	II	35 (64.8)	267 (81.9)	0.006*
	III	19 (35.2)	59 (18.1)	
BMI (kg/m^2^)		25.8±2.5	25.6±2.5	0.574
Abnormal ECG before the surgery		16 (29.6)	58 (17.8)	0.062
Smoking		20 (37.0)	78 (23.9)	0.045*
History of myocardial infarction		13 (24.1)	31 (9.5)	0.005*
History of cerebrovascular disease		1 (1.9)	3 (0.9)	0.46
History of coronary artery surgery		0 (0.0)	25 (7.7)	0.034*
β-blocker		12 (22.2)	112 (34.4)	0.086
Antiplatelet medication		0 (0.0)	87 (26.7)	<0.001*
Hypertension		9 (16.7)	78 (23.9)	0.295
Diabetic mellitus		14 (25.9)	52 (16.0)	0.082
Baseline SPO_2_ (%)		92.3±0.8	92.1±0.7	0.035*
Baseline CO_2_ (%)		27.7±3.3	27.6±3.3	0.813
Baseline cTnI	Positive	5 (9.3)	6 (1.8)	0.011*
	Negative	49 (90.7)	320 (98.2)	
Baseline HIF-1α (ng/mL)		38.9±11.4	33.7±5.4	0.002*
Baseline HO-1 (ng/mL)		32.3±9.1	27.4±2.7	<0.001*

Data are presented as mean ± standard deviation or number (percentage).

### Trends of cTnI, HIF-1α, and HO-1 levels during the perioperative period

**Figure 1:**
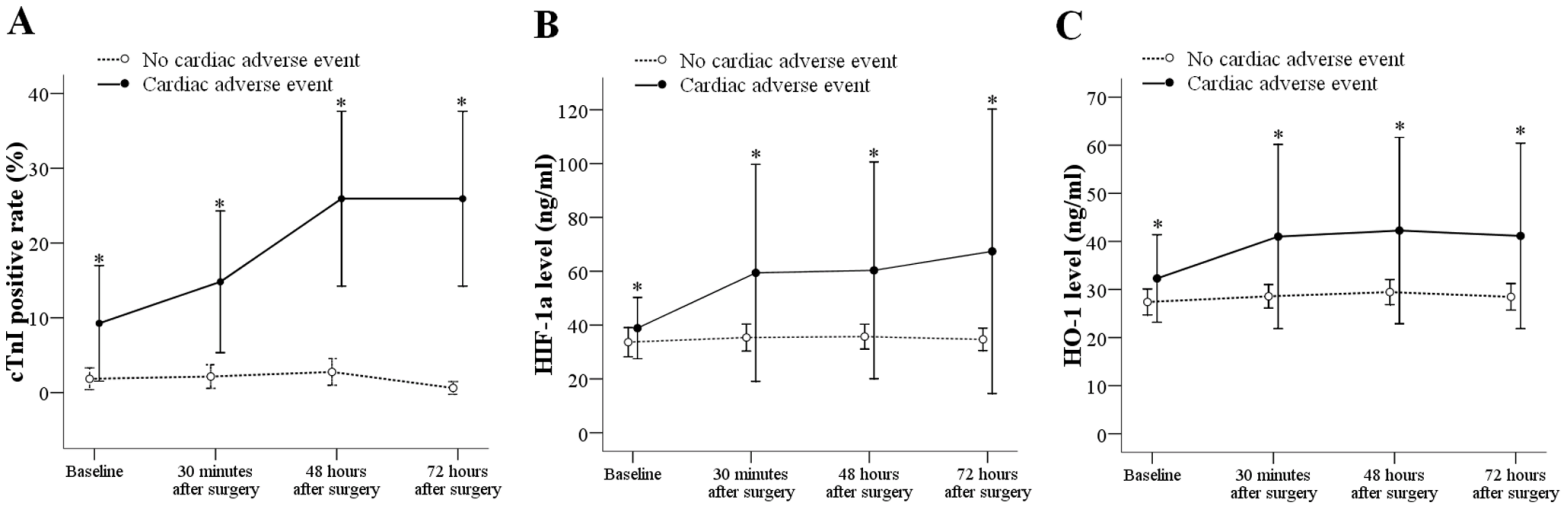
Changes of cTnI, HIF-1α and HO-1 during the perioperative period. The cTnI positive rate (A) and serum HIF-1α (B) and HO-1 (C) levels at the 4 time points (baseline, 30 min after surgery, 48 h after surgery, and 72 h after surgery) from patients with or without adverse cardiac events. *Indicates a significant difference between those with and without an adverse cardiac event. (A) Data were presented as percentage and 95% confidence interval. (B, C) Data were presented as mean and standard deviation.

The perioperative changes of cTnI, HIF-1α, and HO-1 are shown in Figure 1. In the group without adverse cardiac events, the cTnI positive rate was stable during perioperative period (1.8%, 2.1%, 2.8%, and 0.6%). In the cardiac adverse event group, the cTnI positive rate continuously increased from 9.3% at baseline to 25.9% 72 h after the surgery. The cTnI positive rates in the cardiac adverse event group were significantly higher than in the group without cardiac adverse event at each time point (*P*<0.05).

In the group without adverse cardiac events, HIF-1α level was stable during the perioperative period (33.7, 35.4, 35.7, and 34.7 ng/mL). In the cardiac adverse event group, HIF-1α level increased from 38.9 ng/mL at baseline to 59.4 ng/mL 30 min after the surgery, then remained at 60.3 ng/mL 48 h after the surgery, and finally slightly increased to 67.4 ng/mL 72 h after surgery. The HIF-1α levels in the adverse cardiac event group were significantly higher than in the group without adverse cardiac events at each time point (all, *P*<0.05).

In the group without adverse cardiac events, HO-1 level was stable during the perioperative period (27.4, 28.6, 29.5, 28.5 ng/mL). In the cardiac adverse event group, HO-1 level increased from 32.1 ng/mL at baseline to 41.0 ng/mL 30 min after surgery, then remained stable (Fig. 1). The HO-1 levels in the cardiac adverse event group were significantly higher than in the group without adverse cardiac events at each time point (*P*<0.05).

### Factors associated with adverse cardiac events

The results of the univariable and multivariable logistic regression analyses are shown in Table 2. In multivariable analysis, 6 factors (age, race, abnormal ECG before the surgery, history of myocardial infarction, baseline positive cTnI, and baseline HO-1) were found to be independently associated with adverse cardiac events. Controlling for the other 5 variables, the odds of an adverse cardiac event was increased by every 1 year increase in age (odd ratio [OR] 1.39, *P*<0.001), abnormal ECG at baseline (OR 2.27, *P* = 0.048), myocardial infarction history (OR 3.18, *P* = 0.015), and positive baseline cTnI level were associated with an increased likelihood of an adverse cardiac event (OR 8.78, *P* = 0.019), and for every 1 unit increase of HO-1, the odds of an adverse cardiac event increased by 1.30 (*P* = 0.002). Han ancestry was associated with a decreased likelihood of an adverse cardiac event (OR 0.42, *P* = 0.021);

**Table 2:** Risk factors of adverse cardiac events.

		Univariable		Multivariable	
		OR (95% CI)	*P*-value	OR (95% CI)	*P*-value
Age (y)		1.32 (1.18, 1.48)	<0.001*	1.39 (1.21, 1.61)	<0.001*
Gender (female vs. male)		0.59 (0.33, 1.06)	0.08		
Race (Han vs. other)		0.48 (0.27, 0.85)	0.012*	0.42 (0.20, 0.88)	0.021*
NYHA class	I	Reference			
	II	1.85 (0.98, 3.48)	0.056		
	III	1.92 (0.78, 4.72)	0.157		
ASA grade (III vs. II)		2.46 (1.31, 4.59)	0.005*	1.71 (0.75, 3.93)	0.204
BMI (kg/m^2^)		1.03 (0.92, 1.16)	0.573		
Abnormal ECG before the surgery		1.95 (1.02, 3.72)	0.045*	2.27 (1.01, 5.11)	0.048*
Smoking		1.87 (1.02, 3.44)	0.044*	1.87 (0.86, 4.06)	0.115
History of myocardial infarction		3.02 (1.46, 6.23)	0.003*	3.18 (1.26, 8.04)	0.015*
History of cerebrovascular disease		2.03 (0.21, 19.90)	0.543		
History of coronary artery surgery		NA			
β-blocker		0.55 (0.28, 1.08)	0.082		
Antiplatelet medication		NA			
Hypertension		0.64 (0.30, 1.36)	0.243		
Diabetic mellitus		1.84 (0.94, 3.63)	0.076		
Baseline positive cTnI		5.44 (1.60, 18.51)	0.007*	8.78 (1.43, 53.71)	0.019*
Baseline SPO_2_ (%)		1.49 (1.03, 2.17)	0.036*	1.55 (0.98, 2.43)	0.059
Baseline CO_2_ (%)		1.01 (0.93, 1.10)	0.812		
Baseline HIF-1α (ng/mL)		1.10 (1.06, 1.14)	<0.001*	1.01 (0.94, 1.08)	0.822
Baseline HO-1 (ng/mL)		1.27 (1.15, 1.40)	<0.001*	1.30 (1.10, 1.53)	0.002*

CI: confidence interval. NA: the odds ratio was not applicable due to the zero count.

* Indicates a significant influence on the occurrence of an adverse cardiac event.

### Hypoxic marker levels in various cardiac adverse event groups

In the adverse cardiac event group there were 27 cases of acute myocardial ischemia, 12 cardiac arrhythmias, 10 acute myocardial infarctions, and 5 cases of heart failure. There were no significant differences in HIF-1α and HO-1 levels between the groups with various types of adverse cardiac events (data not shown). The associations between cTnI levels and adverse cardiac events type at the 4 time points are shown in Table 3. Before the surgery, no significant difference in cTnI level was observed between the different adverse cardiac event groups. Acute myocardial infarction was associated with a significantly higher cTnI positive rate than acute myocardial ischemia and cardiac arrhythmia at 30 min (60.0% vs. 3.7% and 0%), 48 h (90.0% vs. 3.7% and 0%), and 72 h (90.0% vs. 0% and 0%) after the surgery. Heart failure was associated with a significantly higher cTnI positive rate than acute myocardial ischemia and cardiac arrhythmia at 48 h (80.0% vs. 3.7% and 0%) and 72 h (100% vs. 0% and 0%) after the surgery.

**Table 3:** cTnI levels in the various cardiac adverse event groups.

	Cardiac adverse event group				P-value
	Acute myocardial ischemia (n = 27)	Cardiac arrhythmia (n = 12)	Acute myocardial infarction (n = 10)	Heart Failure (n = 5)	
Baseline cTnI	1 (3.7%)	0 (0.0%)	4 (40.0%)	0 (0.0%)	0.012
cTnI 30 min after surgery	1 (3.7%)	0 (0.0%)	6 (60.0%)* †	1 (20.0%)	<0.001
cTnI 48 h after surgery	1 (3.7%)	0 (0.0%)	9 (90.0%)* †	4 (80.0%)* †	<0.001
cTnI 72 h after surgery	0 (0.0%)	0 (0.0%)	9 (90.0%)* †	5 (100.0%)* †	<0.001

Data were presented as count of positive cTnI and positive rate of cTnI.

* Indicates a significant difference as compared to acute myocardial ischemia group.

† Indicates a significant difference as compared to cardiac arrhythmia group.

## Discussion

This study showed that in elder patients undergoing non-cardiac surgery, higher age, history of myocardial infarction, abnormal ECG before surgery, positive baseline cTnI level, higher baseline HO-1 level and race (non-Han ancestry) were associated with an increased risk of adverse cardiac events. Though multivariable analysis indicated that baseline HIF-1α level was not a predictive factor of having an adverse cardiac event, levels were significantly increased at each postoperative time in the group with adverse cardiac events as compared to the group without adverse cardiac events.

Cardiovascular events are a leading cause of morbidity and mortality in surgical patients, and many scoring systems and biomarkers have been examined to evaluate cardiac risk before non-cardiac surgery. Elevated BNP levels is correlated with left ventricular systolic dysfunction and it has been utilized in the diagnosis of acute coronary syndrome, heart failure, and other cardiac disorders. Research had examined its use as a biomarker for cardiac events in patients undergoing non-cardiac surgery. Novo et al. [14] examined N-terminal fragment of proBNP (NT-proBNP) levels in 82 consecutive patients undergoing elective non-cardiac surgery and found that a pre-operative elevated NT-proBNP level was independently associated with postoperative cardiac events (odds ratio 1.2, 95% confidence interval 1.0–1.4, *P* = 0.01). Similarly, Kim et al. [15] in a study of 163 patients without a history of cardiovascular disease undergoing non-cardiac surgery reported that preoperative BNP levels were significantly higher in patients who experienced postoperative cardiac events than in those who did not. Mercantini et al. [16] studied 205 patients undergoing major abdominal surgery who received routine cardiac risk assessment and preoperative BNP levels, and reported that a preoperative BNP level >36 pg/mL was the only effective predictor of adverse cardiac events. The ongoing DECREASE-VI study is designed to determine the role of NT-proBNP levels in patients undergoing major vascular surgery [17]. Compare to BNP, the role of HO-1 as prognostic factor may be restricted to the cardiac events that derived from ischemic damage.

HIF-1α is present in the cytoplasm, and it is regulated by oxygen level [5], [6]. It is the only oxygen-regulated subunit of HIF-1, and determines HIF-1 activity [5], [6]. When the oxygen concentration is normal, 2 proline residues in the oxygen-dependent degradation domain (ODD) of HIF-1α are hydroxylated by proteolytic enzymes and degrade rapidly [18]. However, the enzyme activity is inhibited in the hypoxic state, resulting in elevated levels of HIF-1α [18]. When the blood oxygen level is normal, HIF-1α has a very short half-life [5], [6].

Previous study has shown that persistent ischemia and hypoxia increase myocardial cell apoptosis [19]. During an acute myocardial infarction, HIF-1α enters into the blood with a subsequent increase in serum level. HIF-1α is the most sensitive marker of acute hypoxia, and study has shown that high expression of HIF-1α has protective effects against acute coronary ischemia [3]. However, while HIF-1α activation within a short time period has a protective effect, sustained levels for a long period of time may promote disease progression [20]. It has been reported that reactive oxygen species play an important role in activating HIF-1α in the normoxic state [21]. Taking into account that HIF-1α levels were increased at the 3 postoperative time points in the group with adverse cardiac events, it is possible that new ischemia may be occurring in CHD patients undergoing non-cardiac surgery due to surgical stress and trauma. In particular, postoperative HIF-1α levels increased most significantly in the adverse cardiac event group within 30 min after surgery.

HO-1 expression is directly regulated by HIF-1α, and our results showed that serum HIF-1α and HO-1 elevated in the similar time points. HO-1 expression has been associated with the quality and morphology of coronary artery plaques [22], [23], and with protection from ischemia reperfusion injury [24]. Changes in serum HIF-1α and HO-1 levels may not only reflect whether perioperative adverse cardiac events will occur in patients with CHD, but also reflect the severity of the adverse cardiac event. In this study, multivariate logistic regression analysis showed that elevated baseline serum HO-1 was an independent risk factor of perioperative adverse cardiac events, suggesting preoperative HO-1 level may be used as a prognostic factor for adverse cardiac events.

The cTnI level is currently the gold standard indicator of myocardial ischemic necrosis. However, its elevation only appears after myocardial necrosis. As a very sensitive cytokine to hypoxia, HIF-1α may reflect ischemia and hypoxia more rapidly than cTnI. In particular, severe hypoxic-ischemic manifestations (such as ECG abnormalities and cTnI level rise) may not appear after treatment with coronary dilator drugs

There are limitations to this study that should be considered. The sample size is relatively small and the study was performed at a single institution. While the time points for blood collection were chosen to be the most representative of the clinical status of patients, a greater number of sampling time points may provide a more accurate analysis of the rise and fall of the analyte levels. The dosages of drugs used by the patients were not recorded. Lastly, the follow-up period for the recording of adverse cardiac events was relatively short.

### Conclusions

The results of this study showed that perioperative HIF-1α and HO-1 levels were increased in elderly patients who underwent non-cardiac surgery and had subsequent adverse cardiac events, and increased baseline HO-1 level was associated with an increased likelihood of having an adverse cardiac event. Thus, determination of preoperative HO-1 levels may aid in identifying patients with in high risk of developing cardiac ischemia.
